# Improvement in Emulsifying Capacity of Goose Liver Protein Treated by pH Shifting with Addition of Sodium Tripolyphosphate and Its Proteomics Analysis

**DOI:** 10.3390/foods11213329

**Published:** 2022-10-23

**Authors:** Yulong Zhang, Yun Bai, Xiaobo Yu, Zhen Li, Peng Wang, Xinglian Xu

**Affiliations:** 1 College of Food Science and Technology, Nanjing Agricultural University, Nanjing 210095, China; 2 Jiangsu Synergetic Innovation Center of Meat Production and Processing, National Center of Meat Quality and Safety Control, Nanjing 210095, China

**Keywords:** goose liver protein, pH shifting, non-enzyme phosphorylation, emulsifying capacity, proteomics

## Abstract

Goose liver isolate treated by pH shifting and pH shifting/non-enzyme phosphorylation with goose liver isolate was used as a control. The functional property differences in the protein and proteins involved in the interfacial layer treated with pH shifting and non-enzyme phosphorylation were studied. Compared with the goose protein isolates (GPIs) at pH 7.0, the GPIs treated by pH shifting was not a good choice to be an emulsifier in a neutral environment, and non-enzyme phosphorylation inhibited the negative effects of pH shifting treatment and improved protein properties. The results of proteomics showed that the identified proteins in the interfacial layer belong to hydrophilic proteins. Non-enzyme phosphorylation increased the abundances of most proteins due to ion strength, including some phosphorylated proteins. Correlation analysis indicated that protein solubility was highly positively related with S_0_, intrinsic fluorescence, total sulfhydryl, free sulfhydryl, A0A0K1R5T3, R0KA48, R0KFP7, U3J1L1, P01989, R0JSM9, and R0LAD1, and was also highly negatively related with particle size and R0M210, R0M714, and R0LFA3. The emulsifying activity index (EAI) demonstrated highly positive correlation with protein solubility, and was correlated with R0JKI4, R0KK84, R0L1Y3, R0LCM7, A0A068C605, and U3IW62.

## 1. Introduction

Animal liver, as an important organ for an animal, is a byproduct in the processing of meat products, and it contains rich proteins, fat, carbohydrates, vitamins, and minerals [1]. Animal liver is not only a nutrient-rich quality protein source but is also a class of important functional proteins. It contains various essential amino acids in which they occupy appropriate proportions for the human body [2] and antioxidant enzymes, such as a large number of superoxide dismutase, which have obvious anti-lipid oxidation [3]. Animal liver proteins show some functional properties, such as antioxidant activity [1], anti-diabetes [4], and anticoagulation activity [5].

Improvement in the functional properties of food proteins could increase their utilization in the food industry. pH-induced modification and phosphorylation are traditional and simple methods used to modify protein properties. A protein solution in an extreme basis or acidic pH value could make proteins unfold, while pH adjusted back to the isoelectric points could make proteins refold. In these two situations, conformational changes in the proteins occur, exposing hydrophobic sites and sulfhydryl groups [6]. After the above pH-shifting extractions, the partially refolded/unfolded protein structure becomes more flexible, and it is able to be adsorbed more readily to interfaces and yield lower interfacial tension [7]. Some studies have shown that globular proteins may be partially unfolded at extreme pH values, forming a dynamic structure specified as the “molten globule” structure [8,9]. The “molten globule” structure could maintain a firm structure (secondary structure), but it shows a tendency to lose part of the tertiary structure [10]. In the “molten globule” state, proteins show enhanced foaming and emulsifying capacities. The pH shifting treatment could effectively enhance plant protein properties [11,12,13], but Fu et al. [14] found that the pH-shifting treatment partly caused denaturation and degradation of myofibril protein extracted from silver carps and increased the surface hydrophobicity, surface sulfhydryl group content, and total sulfhydryl (TSH) group content. Acid-base extraction conditions were reported to affect protein molecular constituents and structures. Acid treatment leads to degradation of macromolecular proteins, and protein constituents hardly change in the base treatment group, whereas goose liver proteins extracted at pH 11.0 showed the best gelation and emulsification properties [15].

Phosphorylation is used to modify protein properties to improve the water/oil absorption capacity in the emulsification during protein processing. According to previous reports, phosphorylation of proteins is a reaction wherein protein side-chain groups (including hydroxyl groups of threonine, tyrosine and serine, ε-NH_2_ of lysine, the nitrogen atom of arginine guanidine group, and one and three nitrogen atoms of histidine imidazole ring) were selectively induced by a great number of phosphate groups [16]. With the increase in negative charges introduced by phosphate groups on the protein molecular surface, proteins could be enhanced with regard to hydration, leading to the improvement of solubility, water and oil retention capacity, and foaming and emulsifying capacities of proteins [17]. Previous studies have also confirmed that phosphorylation is an effective method to improve protein functional properties, such as foamability, emulsification, water and oil absorption capacity, solubility, and heat stability [18,19,20]. With respect to the emulsifying capacity of protein, phosphorylated proteins rapidly move to oil–water interfaces and improve the adsorption of proteins onto the oil–water interfacial layer and the dispersion of oil droplets by increasing the electrostatic repulsion force of droplets due to the induced negative charges of phosphate groups in protein chain residues [21]. However, a study reported that although phosphorylation improved solubility, foaming properties and water holding capacity, it decreased the emulsifying activity of soy protein compared with unmodified protein. Hence, the improvement of protein emulsifying capacity depends not only on the phosphorylation degree [22] but also on the protein structure after the phosphorylation modification [23].

The functional behavior of liver proteins should be explored to introduce them as a low-cost functional ingredients into food products, and it is also helpful to understand their contribution to food products. In this study, acid-base precipitation was performed to separate goose liver proteins from goose liver protein isolate (GPI), and then pH shifting was used to modify the functional properties of GPI. Finally, non-enzyme phosphorylation was introduced to modify the protein functional properties under the condition of the same pH-shifting procedure. Therefore, this research aimed to evaluate the effect of pH shifting on GPI and the improvement of non-phosphorylation on pH shifting, and to identify the proteins involved in the interfacial layer on the basis of proteomics.

## 2. Materials and Methods

### 2.1. Goose Liver

The goose livers in this study were obtained from Sanhua geese (artificial hybridization species), and they were purchased from a local goose product processing factory (Nanjing, China).

### 2.2. Extraction and Treatment

In accordance with the extraction procedure reported by Li [15], the goose livers were minced, weighed, and mixed with distilled water at a ratio of 1:6 and then homogenized using the adjustable large-scale IKA T25 digital Ultra Turrax homogenizer (IKA, Staufen, Germany) in an ice bath. The homogenate above was adjusted to pH 11.00 with 1 mol/L sodium hydroxide to dissolve the proteins, filtered with a four-layer gauze, precipitated at pH 5.50 with 1 mol/L hydrochloric acid, and finally dissolved at pH 7.00 to obtain GPIs (GP). Due to the improving effects of pH shifting and phosphorylation on protein functions, double pH shifting and non-enzyme phosphorylation were performed as follows. The GP was dissolved in distilled water at a ratio of 1:6 at pH 11.00, precipitated at pH 5.50, and finally dissolved at pH 7.00 to obtain modified extract (PSGP). The GP was dissolved in distilled water at a ratio of 1:6 at pH 11.00 with an addition of sodium tripolyphosphate by the amount of 1% of the dry weight of the goose liver proteins to be inoculated at 40 °C for 50 min to reach non-enzyme phosphorylation, precipitated at pH 5.50, and finally dissolved at pH 7.00 to obtain the phosphorylated extract (PPSGP).

### 2.3. Measurement of Protein Solubility

Protein solubility was performed in accordance with Chen’s method [24]. In brief, aqueous solutions of goose liver protein samples (10 mg/mL) were prepared in distilled water at a final pH of 7.00–12.00. The protein dispersions were stirred at an ambient temperature for 30 min, centrifuged at 10,000× *g* for 20 min, and filtered into tubes. The protein contents of the supernatants were measured in accordance with the Bradford method by using bovine serum albumin (BSA) as a standard. The protein solubility was expressed as a percentage of total protein content.

### 2.4. Determination of Surface Hydrophobicity

Surface hydrophobicity was measured using the method of Chen et al. [24]. Protein samples were dissolved in 20 mmol/L phosphate buffer (pH 7.00) and diluted to a final concentration of 1 mg/mL. Protein solution (1 mL) was added to 2 mL tubes, and 5 μL of 8-anilino-1-naphthalenesulfonic acid (ANS, 15 mmol/L in 0.1 mol/L phosphate buffer, pH 7.00) solution was added to each tube in the darkness. Then, all of the mixed solutions were stored at 25 °C for 20 min in the dark and centrifuged at 4 °C and 10,000× *g* for 10 min. The fluorescence intensity (FI) of each supernatant was determined using a SpectraMax microplate reader (SpectraMax M2e, Molecular Devices, Sunnyvale, CA, USA) at a constant excitation wavelength of 375 nm with a step size of 10 nm, integration every 1 s, and emission wavelength between 410 and 600 nm. Protein solution (1 mg/mL) was diluted with 20 mmol/L phosphate buffer (pH 7.00) to final concentrations of 0.2, 0.4, 0.6, and 0.8 mg/mL. Then, the FI was determined at an excitation wavelength of 375 nm and an emission wavelength obtained from the scanning fluorescence spectrum with an excitation wavelength of 375 nm and an emission wavelength between 410 and 600 nm. The initial slope of the FI versus protein concentration was used as the index of protein hydrophobicity (S_0_).

### 2.5. Intrinsic Fluorescence Analysis

Intrinsic fluorescence was carried out in accordance with Chen’s method [24], with slight modifications. The emission spectra of the clarified soluble liver proteins from different treatments (0.5 mg/mL) were recorded from 320 nm to 420 nm with a constant excitation wavelength of 295 nm, a step size of 1 nm, and integration every 1 s by using the SpectraMax microplate reader (SpectraMax M2e, Molecular Devices, Sunnyvale, CA, USA).

### 2.6. Sulfhydryl Content

Sulfhydryl content was measured in accordance with the method of Jiang et al. [25]. Protein samples were dissolved in phosphate buffer (pH 7.00, containing 0.6 mol/L potassium chloride and 8 mol/L urea) and diluted to a final concentration of 1 mg/mL. Protein solution (0.5 mL) was extracted from each tube and added to 4.5 mL of phosphate buffer (no urea). Then, 20 μL of Ellman’s reagent (5,5′-dithio-bis-2-nitrobenzoic acid, DTNB, 10 mmol/L) was added into each tube (distilled water as the blank). The mixed solutions were stored at 4 °C for 1 h in the dark and then centrifuged at 5000× *g* for 10 min to determine the absorbance at 412 nm by using the SpectraMax microplate reader (SpectraMax M2e, Molecular Devices, Sunnyvale, CA, USA). Reactive sulfhydryl content was measured using the same system in the absence of urea. The TSH content and reactive sulfhydryl content were calculated using the following equation:

$$\mathrm{Sulfhydryl}\;\mathrm{content}\;(\mu \mathrm{mol}/g)=\frac{10^{6}\times A_{412}\times D}{1.36\times 10^{4}\times C},$$

where 1.36 × 10^4^ is the molar absorptivity (M^−1^·cm^−1^); 10^6^ originates from the conversions from molarity to micromolarity and from mg solids to g solids (Ellman, 1959); A_412_ is the absorbance at 412 nm; D is the dilution factor; and C is the protein concentration (mg/mL).

### 2.7. Emulsifying Properties

The emulsifying activity index (EAI) and emulsion stability (ES) of GPI were measured in accordance with Yan et al. [26]. Proteins samples were dissolved in distilled water at a final concentration of 35 mg/mL and adjusted to pH 7.00. GP isolate solution (3 mL) and 7 mL of soybean oil were mixed in a beaker and then homogenized at 10,000 rpm for 30 s by using a T25 digital Ultra Turrax homogenizer (IKA, Staufen, Germany). Immediately, 100 μL of the emulsion from the bottom of the beaker was added to 9.90 mL of 0.10% sodium dodecyl sulfate (SDS) and mixed, and the emulsion absorbance at 500 nm was determined using the SpectraMax microplate reader (SpectraMax M2e, Molecular Devices, Sunnyvale, CA, USA). The height of the emulsion was immediately measured at 0 min. After 4 days in the 4 °C room, the height of the emulsion was measured again. The EAI and ES were calculated using the equations below:

$$\mathrm{EAI}\;(m^{2}/g)=\frac{2\;\times \;2.303\;\times \;A_{0}\;\times \;D}{C\;\times \;\varphi \;\times \;10000};\;\mathrm{ES}\;(\%)=(1-\frac{H_{0}\;-\;H_{4}}{H_{0}})\times 100,$$

where D is the dilution factor; C is the initial protein concentration, mg/mL; φ is the fraction of soybean oil, %; A_0_ is the absorbances of the diluted emulsion at 0 min; H_0_ is the height of the emulsion at 0 min, mm; and H_4_ is the height of the emulsion after 4 days of being stored in 4 °C room, mm.

### 2.8. Particle Size

For determination of the particle size of the protein emulsion, 100 μL of the emulsion from the bottom of the beaker above was added to 9.90 mL of 0.10% SDS and mixed. The particle-size distribution of the sample was determined via dynamic light scattering [27,28] using a Zetasizer Nano ZS 90 (Malvern Instrument Ltd., Great Malvern, UK) equipped with a 4 mW He-Neon laser (λ = 633 nm). Protein solutions (1 mg/mL) were placed in a 1 cm path-length quartz cuvette for the measurement of particle size with a detection angle of 90° at 25 °C. The hydrodynamic diameters of protein particles were estimated from the autocorrelation function through the cumulant method on the basis a single exponential fit of the autocorrelation function to obtain the mean particle size (Z-average diameter). In accordance with the scattering intensity, the distributions of scattering particle size were monitored. The polydispersity index (PDI) value was used as the measurement of the breadth of the size distribution.

### 2.9. Proteomic Analysis

#### 2.9.1. Extraction of Emulsion-Layer Protein

An emulsion sample was centrifuged at 4 °C and 10,000× *g* for 10 min to collect the emulsion layer. The emulsion layer was then mixed with three quantities of Tris buffer (0.1 mol/L, pH 7.00, containing 2% SDS) by using a T25 digital Ultra Turrax homogenizer (IKA, Germany), stored at −80 °C for 24 h, and thawed at 4 °C until the frozen sample was completely thawed. This procedure was performed until the emulsion layer was fully broken. Subsequently, sample solutions were centrifuged at 2000× *g* for 30 min to collect the supernatant, added to an equal quantity of Tris-saturated phenol (Shanghai Yuanye Bio-Technology Co., Ltd., Shanghai, China), and shaken for 15 min at room temperature to collect the phenol layer. The solution of the phenol layer was added with four quantities of pre-cooled 0.1 mol/L ammonium acetate-methanol for protein precipitation at −20 °C overnight and centrifuged at 4 °C and 2500× *g* for 10 min to obtain the protein precipitate. The precipitate was washed with pre-cooled 0.1 mol/L ammonium acetate-methanol, precipitated at −20 °C for 3 h, and centrifuged to obtain the precipitate. This step was repeated twice. The proteins were dissolved in 2 mL of 50 mmol/L ammonium hydrogen carbonate and quantified with a BCA protein assay kit (Bio-Rad, Berkeley, CA, USA).

Samples (containing 100 µg protein) were pooled into EP tubes, added to dithiothreitol (Promega, Madison, WI, USA) at a final concentration of 10 mmol/L, placed in a 50 °C water bath for 1 h, added to iodoacetamide solution at a final concentration of 50 mmol/L, and placed in the dark for 30 min. Subsequently, the samples were placed into 10 kDa ultrafiltration tubes, added to 200 µL of 50 mmol/L ammonium hydrogen carbonate, centrifuged at 2500× *g* twice, and finally dissolved in 100 µL of 50 mmol/L ammonium hydrogen carbonate.

#### 2.9.2. Protein Digestion

Protein digestion was performed in accordance with Jorge’s method [29], with some modifications. First, 1 µg/µL of trypsin was added into the protein sample through an addition of 1 µg trypsin/100 µg proteins to digest in a 37 °C water bath for 4 h. Then, it was added to 1 µL trypsin again to digest for 12 h. Afterwards, the digestive juice was centrifuged at 5000× *g* twice with an addition of 200 µL of 50 mmol/L ammonium hydrogen carbonate to collect peptides for vacuum drying. The peptides of the sample were desalted with sep-Pak C18 cartridges (Waters sep-Pak C18 cartridges, 1 cc/100 mg, Waters, Milford, MA, USA) before freeze drying. The peptide mixture was labeled in accordance with the protein treatment samples, dissolved in 0.1% formic acid (FA, in distilled water), and analyzed by nanoliquid chromatography-tandem mass spectrometry (nano LC/MS).

#### 2.9.3. Protein Identification by MS

An AcclaimTM PepMapTM C18 LC column with a 2 µm particle size and a 100 Å pore-sized C18 capillary (250 mm × 75 µm, Thermo Scientific, Bremen, Germany) was washed with mobile phase A (0.1% FA in water) and mobile phase B (0.1% FA in acetonitrile) for 6 min before the sample injection. Peptides were eluted using a gradient of 5% mobile phase B for 5 min, 5–30% mobile phase B for 90 min, 30–80% mobile phase B for 10 min, 80–5% mobile phase B for 0.1 min, and 5–0% mobile phase B for 14.9 min. The flow rate was 300 nL/min, the column temperature was 30 °C, and the injection volume was 2 µL. Each sample was analyzed three times.

The Q Exactive mass spectrometer (Thermo Scientific) was used in data-dependent acquisition mode for data acquisition. A full-scan MS scan [automatic gain control (AGC) set to 5 × 10^5^ ions with a maximum fill time of 50 ms] was carried out using a mass spectrometer equipped with a nanospray LTQ Orbitrap over 350–1600 *m/z* with a resolution of 70,000 (200 *m/z*) for the first-level scan. Subsequently, the 10 most abundant peaks were selected for MS/MS by using higher-energy C-trap dissociation and a scan of 17,500 resolutions (AGC set to 2.0 × 10^5^ ions with a maximum fill time of 150 ms) over 100–2000 *m/z* with a selected window of 2 Da and a normalized collision energy of 27%. The peptide charges were +2, +3, and +4. The program used a 60 s-dynamic exclusion window to avoid repeated selection of peptides for MS/MS. Raw files were processed with MaxQuant (version 1.5.8.3). The nano LC-MS/MS data were submitted to a local server for protein identification via MaxQuant (version 1.5.8.3) using a custom database of Anatidae (waterfowl) downloaded from Uniprot and protein quantitation based on iBAQ.

### 2.10. Statistical Analysis

All experiments were performed three times on different batches. Each assay was carried out three times at least. All data were subjected to ANOVA using the SAS software (SAS 8.2. SAS Inst. Inc., Cary, NC, USA). Duncan’s multiple range comparison was used to determine significant differences for emulsifying properties (*p* < 0.05) between means. PyMOL (version 2.2.0, Schrödinger Inc., New York, NY, USA) was used to produce high-quality three-dimensional images of the proteins. R programming language (version 3.4.4) with a pheatmap package was used to construct the heatmap.

## 3. Results and Discussion

### 3.1. Protein Solubility

**Figure 1:**
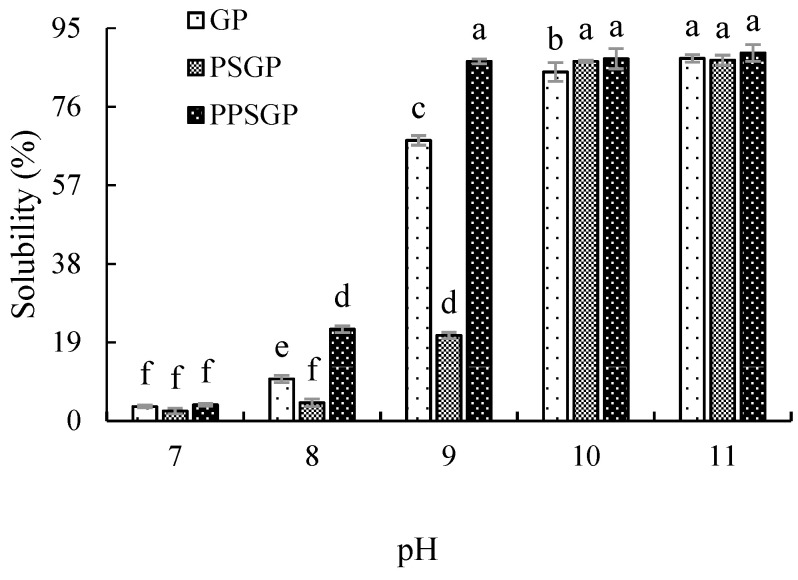
Protein solubility of GP, PSGP, and PPSGP. Different letters indicate significant differences.

An increase in protein solubility could improve protein properties, so protein solubility is considered as one of the most practical factors of the functional properties of proteins [30], and excellent protein solubility is a precondition for the application of a protein as an emulsifier [31]. In the present study, the protein solubility of GP, PSGP, and PPSGP was varied in different pH values (Figure 1). The protein solubility of PPSGP was the highest in the pH range of 7.00–9.00, whereas that of PSGP was the lowest. With the continuous increase in pH, the protein solubility of GP, PSGP, and PPSGP increased, and no significant difference was found in the protein solubility between each sample (GP, 87.73%; PSGP, 87.29%; and PPSGP, 89.01%) at pH 11.00. Under low pH, the solubility of the protein was affected by increased particle size, buried polar groups, and weakened interaction with water [32]. The unfolding and refolding of protein molecules in the pH-shifting process may destroy the hydrophobic interaction and van der Waals forces between protein molecules, leading to an increase in solubility [33]. As a consequence, the interactions between protein and water molecules were improved, resulting in increased solubility. Jiang et al. [34] reported that pH shifting altered the solubility characteristics and thermal stability of soy protein isolate and its globulin fractions in different pH conditions. Double alkaline pH shifting changed the solubility of goose liver proteins at different pH levels, and this treatment made proteins unfold and fold again, thus altering the hydrophobic interaction and van der Waals forces between protein molecules and the interactions between protein and water molecules. The addition of sodium tripolyphosphate increased the solubility of PPSGP when compared to GP and PSGP, which indicated that non-enzyme phosphorylation decreased the negative effect of double pH shifting on goose liver proteins. Sodium tripolyphosphate reacted with protein molecules at pH 11.00, leading to the alteration of ion strength and protein structure. The inorganic phosphate group of some phosphorylation reagents, such as sodium tripolyphosphate, phosphorus oxychloride, and sodium trimetaphosphate, could react with the -OH side chain groups of tyrosine, threonine, and serine; -N of arginine guanidine group; 1 and 3-N of histidine imidazole ring; and ε-NH_2_ of lysine, consequently forming C-O-P structure or C-N-P structure [35]. The introduction of phosphate groups created a strong electrostatic repulsion between protein molecules, enhancing water solubility, water-holding, and oil-binding capacities and foaming and emulsifying protein properties [23,36]. These findings explained the influence of protein phosphorylation on the functionality of goose liver protein.

### 3.2. Surface Hydrophobicity

**Figure 2:**
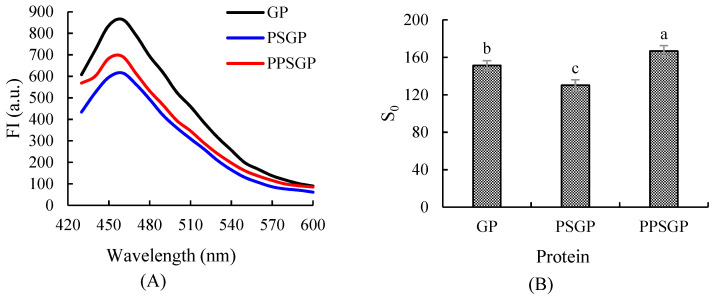
Fluorescence spectroscopy of GP isolate for surface hydrophobicity and an index of protein hydrophobicity (S_0_). (**A**) represented surface hydrophobicity; (**B**) represented S_0_. Different letters indicate significant differences.

Surface hydrophobicity could reflect the degree of protein unfolding and the exposure of hydrophobic groups on the surface, which influences the hydrophobic interaction between proteins and their emulsifying, foaming, and gelling capacities [37]. Figure 2A shows the surface hydrophobicity of GP, PSGP, and PPSGP by fluorescence spectroscopy. The maximum fluorescence emission was found at 460 nm for all groups. Good emulsifying activity depends on the balance between hydrophobic and hydrophilic groups [38]. Different treatments interfered with the balance of surface hydrophobicity, which increased or decreased. PSGP showed lower FI due to changes in goose liver proteins than GP and PPSGP. Double pH shifting made protein molecules shrink and refold, leading to protein denaturation. Under unstable tertiary and quaternary structures, the hydrophobic groups that were previously exposed on the protein molecule surface were buried in the interior regions of the protein in aqueous environment because of denaturation. Less ANS had access to bind with previous non-polar parts of protein molecules. Thus, the surface hydrophobicity of proteins decreased. This variation in fluorescence may have also resulted from different energy transfer efficiencies between tryptophan (Trp) and tyrosine [39] or the enclosure of the chromophores. As shown in Figure 2B, PPSGP showed the highest S_0_. Non-enzyme phosphorylation of goose liver proteins significantly enhanced the surface hydrophobicity (*p* < 0.05). During the process of non-enzyme phosphorylation treatment, the phosphate groups were bound to proteins, which changed their surface charge [40,41], thereby contributing to the change in protein surface hydrophobicity.

### 3.3. Intrinsic Fluorescence

**Figure 3:**
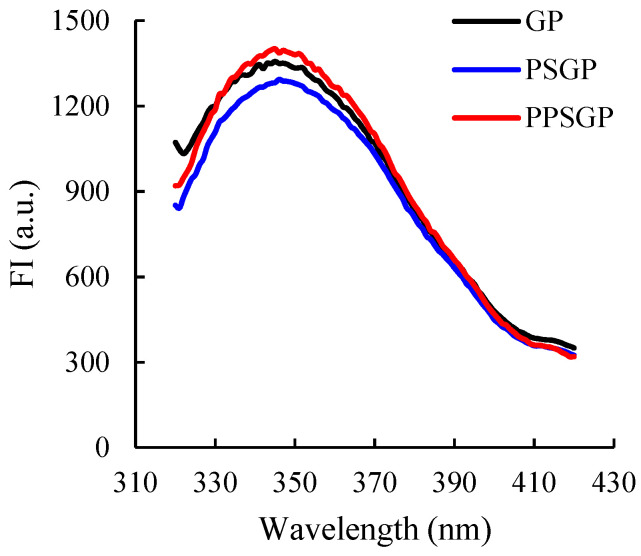
Fluorescence spectroscopy of GP isolate for intrinsic fluorescence.

The intrinsic fluorescence spectrum was generally used to characterize the changes in tertiary structure of proteins due to the sensitivity of intrinsic fluorescence of aromatic amino acid residues to the polarity of microenvironments [33,42]. It is mainly attributable to Trp. Thus, fluorescence spectroscopic analysis is suitable for the evaluation of changes in the tertiary structure of GPI. As shown in Figure 3, the maximum emission wavelength (λmax) values of GP, PSGP, and PPSGP were 345, 346, and 345 nm, respectively. Compared with the FI of GP, that of PSGP remarkably decreased. The double pH shifting process altered the compact structure of goose liver proteins, thereby changing the extent of the exposure of chromophores to the solvent [9]. This result was consistent with the change trend in surface hydrophobicity (Figure 2), which showed less exposure of hydrophobic groups due to the refolding of GPI. Moreover, partial refolding of protein molecules due to the double pH shifting process may increase the internal quenching, which also contributed to the decrease in FI [43]. PPSGP showed the highest intrinsic FI at 345 nm. Phosphorylation could introduce charged and hydrophilic groups in the side chain of amino acids and change ion strength, altering the structural conformation of proteins [36]. Therefore, the addition of sodium tripolyphosphate and the introduction of phosphate groups changed the protein functionality of GPI and increased its FI. The introduction of phosphate groups and ion strength also led to higher FI of PPSGP when compared to that of GP.

### 3.4. Sulfhydryl Content

**Figure 4:**
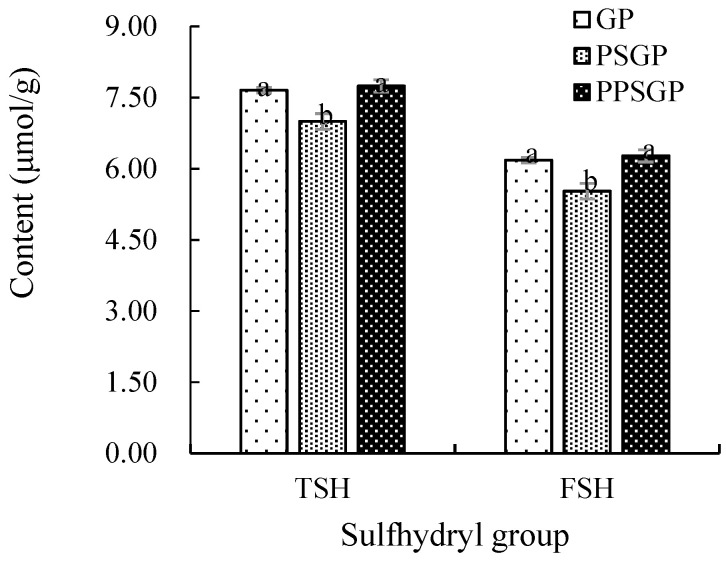
Sulfhydryl content of GP isolate. TSH: total sulfhydryl; FSH: free sulfhydryl. Different letters indicate significant differences.

The TSH and free sulfhydryl (FSH) contents of GP, PSGP, and PPSGP are shown in Figure 4. The TSH content of GP was 7.65 μmol/g, and this parameter was significantly affected by pH-shifting treatment, which seemed to be related to protein refolding. PSGP had lower TSH content (7.00 μmol/g) than GP due to double pH shifting treatment. Non-enzyme phosphorylation increased the TSH content and eliminated the difference between GP and PPSGP (7.74 μmol/g). The changes in the FSH content of GPI were similar to those in the TSH content. Sulfhydryl groups belong to weak secondary bonds, and they are helpful for maintaining the tertiary structure of proteins [44]. Changes in sulfhydryl content could be used to reflect the alteration degree of tertiary and quaternary structures, indicating protein denaturation to some extent [45]. Therefore, a decrease in the sulfhydryl content of PSGP revealed that double pH shifting resulted in protein denaturation, causing refolding and aggregation of GPI and burying sulfhydryl groups outside the protein [12]. In addition, the electrostatic repulsion between proteins was relatively small near the isoelectric point of proteins, so protein particles tended to aggregate and the particle size increased, thus impeding the exposure of sulfhydryl group [46]. Non-enzyme phosphorylation increased the sulfhydryl content of GPI, and similar results were observed in egg white protein [19], soy protein isolate [47], and perilla protein isolate [48]. The introduction of phosphate groups and ion strength inhibited the negative effect of double pH shifting, altering protein structure and the solubility, and finally led to the exposure of sulfhydryl groups, which explained no significant difference between GP and PPSGP.

### 3.5. Emulsifying Property

**Figure 5:**
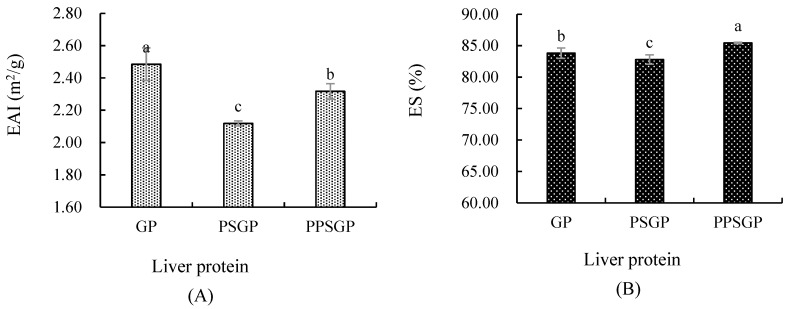
Emulsifying activity and emulsion stability of goose liver protein isolate. (**A**) EAI, emulsifying activity index; (**B**) ES, emulsion stability. Different letters indicate significant differences.

Emulsifying properties characterize the ability of a protein to adsorb to the oil–water interface, and they are commonly assessed by EAI and ES. EAI was defined as the interfacial area a unit weight of proteins produced, and it was used to indicate the emulsifying activity of proteins, indicating the relative surface coverage of a protein on an oil droplet within a dilute emulsion. The emulsifying activity of proteins showed rapid absorption at oil–water interfaces during formation of the emulsion [49]. The EAI of GPI is shown in Figure 5A. GP had the significantly highest EAI (*p* < 0.05), followed by PPSGP (2.32 m^2^/g) and PSGP (2.12 m^2^/g). Non-enzyme phosphorylation effectively inhibited the negative effect of pH-shifting treatment on protein EAI. Similar findings were observed in the ES of GPI (Figure 5B). ES indicated the relative stability of the emulsion after a pre-determined time [50], and it was related to continuous and dispersed phases. The formation of a stable emulsion may be attributed to protein molecules remaining at the interface after absorption for the stability of oil droplets [51]. Low β-sheet content could impair protein interactions, resulting in inferior emulsion properties, and the goose liver proteins modified by different treatments showed different emulsifying properties because double pH shifting and non-enzyme phosphorylation led to differences in goose liver protein structures [48,52]. As shown in Figure 5, double pH shifting reduced the EAI and ES values of GPI, indicating that pH-shifting-treated GPI was less capable of being adsorbed to the oil–water interface. The formed emulsion had worse stability than the control GP, and PPSGP had the highest ES. Protein solubility and molecular flexibility at the interface also affected emulsifying properties [26]. Thus, the reduced or increased emulsifying properties of GPI were well correlated with reduced solubility and the changes in structural properties [33].

### 3.6. Particle Size

The particle sizes of GPI and GPI emulsion are shown in Table 1. The average particle size of the control GP was 2614.33 nm, and double pH-shifting treatment significantly increased the particle size of GPI (*p* < 0.05). This result suggested that double pH shifting changed the interactions between protein molecules and induced protein aggregation [12,53]. Thus, the particle size of GPI increased from 2614.33 nm to 2838.67 nm. The interactions between proteins and water molecules were weakened, and protein aggregates with bigger sizes became more insoluble. Therefore, the protein solubility in water decreased. Bigger protein aggregates also contribute to decreased water solubility due to a smaller interaction area between protein and water molecules [38]. Furthermore, the increased protein size reduced the adsorption rate of protein to the oil–water interface, thus decreasing its emulsifying ability [54]. The addition of phosphate group modified the protein structure and function, thus obviously reducing the particle size of GPI. As for GPI emulsion, the particle size of GP emulsion was significantly the smallest (*p* < 0.05), followed by that of PPSGP emulsion (3172.33 nm), whereas that of PSGP emulsion was the biggest (*p* < 0.05). A bigger particle size of PSGP emulsion could result from the solubility and particle size of protein and protein conformation, and it also indicated the stability of protein emulsion.

### 3.7. Proteomic Analysis for Proteins Involved in Interfacial Layer

**Figure 6:**
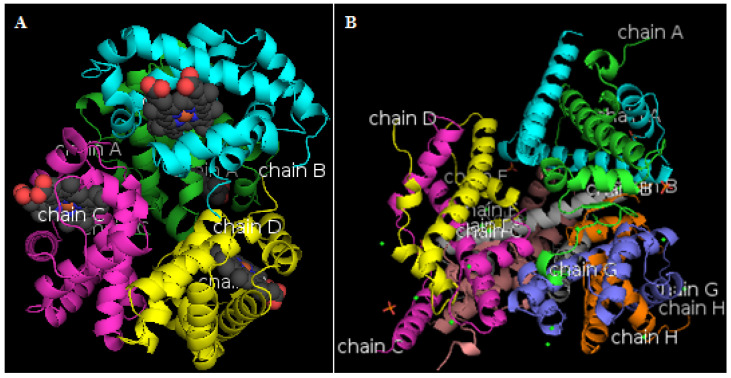
Structures of hemoglobin subunit alpha-A and ATP synthase subunit beta. (**A**) hemoglobin subunit alpha-A; (**B**) ATP synthase subunit beta.

Through proteomics identification, 1054, 1072, and 1071 kinds of proteins were identified in GP, PSGP, and PPSGP interfacial layers, respectively. As shown in Table 2, Table 3 and Table 4, similar ranges of molecular weight (MW), theoretical isoelectric point (theoretical pI), and grand average of hydropathicity (GRAVY) were found among the top 10 proteins in the GP, PSGP, and PPSGP groups. The differences in these top 10 proteins were mainly attributed to changes in protein structure and functionality. From the top 10 proteins of the highest signal strength in each group, the relative quantity of hemoglobin subunit alpha-A was the highest, and that of ATP synthase subunit beta was also higher. The structures of these two proteins with low molecular weight are shown in Figure 6. Hemoglobin subunit alpha-A consists of four chains, and ATP synthase subunit beta consists of eight chains. A previous study revealed that the secondary structure properties and surface hydrophobicity of a protein affect its emulsifying property [52]. Goose liver proteins extracted from the interfacial layer may have low molecular weight, high surface hydrophobicity, and other properties contributing to the improvement of emulsifying properties. Some proteins were degraded to disable the proteins to stably be adsorbed onto oil surface during the pH-shifting process, thus impairing the emulsifying properties of proteins [55]. The loss of major native secondary structures at the expense of the formation of β-sheets could promote the crosslinking between protein particles, possibly contributing to the formation of a more stable protein interfacial film coated on the oil surface [56]. Thus, pH shifting changed the secondary structures and the exposure of protein hydrophobic groups, leading to the adsorption of proteins onto the oil–water interfacial layer and the dispersion of oil droplets, consequently affecting the kinds and quantities of goose liver proteins in the interfacial layer.

The phosphorylation site, pI, GRAVY, and abundance of phosphorylated proteins in each group are shown in Table 5. Phosphorylation occurred at the side chains of threonine, serine, tyrosine, and lysine of goose liver proteins. Phosphorylated proteins were upregulated or downregulated due to double pH-shifting treatment and protein phosphorylation. Glyceraldehyde-3-phosphate dehydrogenase, glutathione S-transferase, and A8RRQ6 (isocitrate dehydrogenase) were highly enriched in each group, suggesting that these proteins had high emulsifying capacities. The pH-shifting treatment led to a decrease in the abundances of glyceraldehyde-3-phosphate dehydrogenase, glutathione S-transferase, isocitrate dehydrogenase, R0JSM9 (heat shock protein HSP 90-alpha), R0LAD1 (ribosomal protein), R0LCM7 (sulfotransferase), U3IQ39 (heterogeneous nuclear ribonucleoprotein A3), and A0A068C605 (elongation of very long chain fatty acid protein 5), especially U3IW62 (RNA helicase). pH shifting changed the secondary structures and the exposure of protein hydrophobic groups, leading to the adsorption of proteins onto the oil–water interfacial layer and the dispersion of oil droplets, consequently affecting the kinds and quantities of goose liver proteins involved in the emulsion layer. Moderate exposure of protein hydrophobic groups could promote the interaction of the protein molecule and the oil molecule to facilitate more proteins to coat on the surfaces of oil molecules, consequently leading to a decrease in the oil–water interfacial tension, together with the enhancement of the emulsifying stability [57]. This finding explained why the pH shifting treatment also resulted in an increase in the abundances of isocitrate dehydrogenase, R0LFA3 (coatomer subunit beta), and U3IFD3 (myosin heavy chain 11). Protein phosphorylation increased the abundances of glyceraldehyde-3-phosphate dehydrogenase, glutathione S-transferase, isocitrate dehydrogenase, heat shock protein HSP 90-alpha, ribosomal protein, sulfotransferase, and elongation of very long chain fatty acid protein 5 due to the ion strength change and the introduction of phosphate groups. The induction of phosphate groups in protein chain residues could change the negative charges of goose liver proteins, leading to the improvement of the adsorption of proteins onto the oil–water interfacial layer and the dispersion of oil droplets [21]. Consequently, the phosphorylated proteins could be adsorbed onto the oil–water interfacial layer.

### 3.8. Correlation Analysis

**Figure 7:**
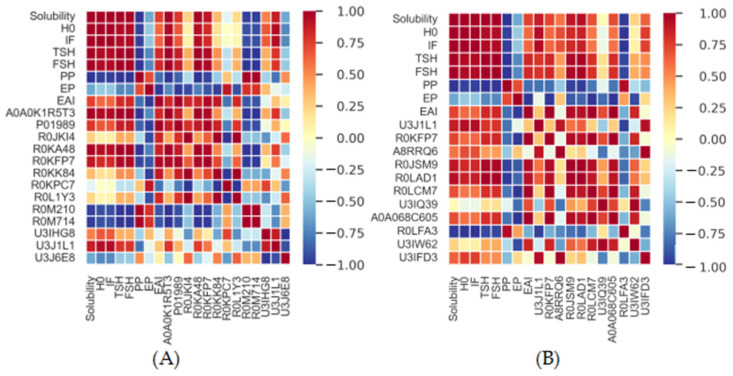
Correlation analysis. IF: intrinsic fluorescence; PP: protein particle; EP: emulsion particle. (**A**) The top 10 proteins of the highest signal strength; (**B**) Phosphorylated proteins.

The correlations between the parameters above and the top 10 proteins and phosphorylated proteins are shown in Figure 7. For protein properties, the solubility of GPI was highly and positively related to S_0_, IF (peak value), TSH, and FSH and highly and negatively related to particle size. The EAI was positively related to solubility, TSH, FSH, S_0_, and IF (peak value of intrinsic fluorescence) and highly and negatively related to particle size. As for the top 10 proteins of the highest signal strength (Figure 7A) and phosphorylated proteins (Figure 7B), the solubility was highly and positively correlated with A0A0K1R5T3, R0KA48, R0KFP7, U3J1L1, P01989, R0JSM9, R0LAD1, R0LCM7, and A0A068C605 and highly and negatively related with R0M210, R0M714, and R0LFA3. The EAI showed a high relationship with A0A0K1R5T3, P01989, R0JKI4, R0KA48, R0KFP7, R0KK84, R0L1Y3, R0JSM9, R0LAD1, R0LCM7, A0A068C605, U3IQ39, and U3IW62. However, the EAI was highly negatively correlated with R0M210 and R0M714. Therefore, pH shifting and non-enzyme phosphorylation could change the original natural structures and composition of GP, thus affecting its functional properties.

## 4. Conclusions

The pH-shifting treatment and protein phosphorylation contributed to the physicochemical properties of GPI proteins. The goose liver protein treated with pH shifting exhibited worse potential as an emulsifier in neutral environments than GP, and pH shifting induced worsened solubility, structural changes, decreased FSH content, worsened emulsifying activity and ES, and enlarged particle sizes of GPI and oil droplets. Non-enzyme phosphorylation could effectively inhibit the negative effects of the pH-shifting treatment, thus improving the protein properties. The identified proteins in the interfacial layer were small-molecular-weight proteins with theoretical isoelectric points of 5–12, and most of them belonged to hydrophilic proteins. Protein solubility was highly and positively related with S_0_, IF, TSH, and FSH and A0A0K1R5T3, R0KA48, R0KFP7, U3J1L1, P01989, R0JSM9, and R0LAD1 and highly and negatively related with particle size and R0M210, R0M714, and R0LFA3. The EAI showed a highly positive correlation with protein solubility and physicochemical parameters. It was also positively correlated with R0JKI4, R0KK84, R0L1Y3, R0LCM7, A0A068C605, and U3IW62.

## Figures and Tables

**Table 1:** Particle sizes of GP and GP emulsion.

Sample		Particle Size (nm)	PDI
GPI	GP	2614.33 ± 48.69 ^b^	0.22 ± 0.07 ^a^
	PSGP	2838.67 ± 56.37 ^a^	0.24 ± 0.06 ^a^
	PPSGP	2575.33 ± 69.15 ^b^	0.02 ± 0.02 ^b^
GPI emulsion	GP	2668.33 ± 229.84 ^c^	0.18 ± 0.02 ^b^
	PSGP	3497.00 ± 132.35 ^a^	0.33 ± 0.15 ^a^
	PPSGP	3172.33 ± 232.00 ^b^	0.45 ± 0.09 ^a^

Means in the same column with different subscript letters as to the same parameter show significant difference (*p* < 0.05, n = 3). PDI is “polydispersity index”.

**Table 2:** Rank of the top 10 proteins of the highest signal strength in the GP group.

Protein ID	Description	MW (kDa)	pI	GRAVY	iBAQ Value
P01989	Hemoglobin subunit alpha-A	15.44	8.54	0.068	5.17 × 10^9^
R0KA48	Histone H4	11.37	11.36	−0.521	1.79 × 10^9^
U3J6E8	Apovitellenin-1	9.49	9.34	0.24	1.75 × 10^9^
R0KK84	ATP synthase subunit beta, mitochondrial	13.80	5.32	0.117	1.68 × 10^9^
U3J1L1	Glyceraldehyde-3-phosphate dehydrogenase	35.84	8.87	−0.065	1.50 × 10^9^
R0KFP7	Glutathione S-transferase	25.34	8.87	−0.319	6.70 × 10^8^
U3IHG8	Fructose-bisphosphate aldolase	39.27	8.74	−0.266	6.59 × 10^8^
R0L1Y3	60 kDa heat shock protein, mitochondrial	61.03	5.61	−0.069	5.83 × 10^8^
R0JKI4	Glutathione-requiring prostaglandin D synthase	22.46	6.83	−0.215	4.56 × 10^8^
A0A0K1R5T3	ADP/ATP translocase	32.79	9.73	0.092	4.04 × 10^8^

q-value is lower than 0.0001.

**Table 3:** Rank of the top 10 proteins of the highest signal strength in the PSGP group.

Protein ID	Description	MW (kDa)	pI	GRAVY	iBAQ Value
P01989	Hemoglobin subunit alpha-A	15.44	8.54	0.068	2.52 × 10^9^
U3J6E8	Apovitellenin-1	9.49	9.34	0.24	1.67 × 10^9^
U3J1L1	Glyceraldehyde-3-phosphate dehydrogenase	35.84	8.87	−0.065	1.39 × 10^9^
R0KA48	Histone H4	11.37	11.36	−0.521	1.35 × 10^9^
R0KK84	ATP synthase subunit beta, mitochondrial	13.80	5.32	0.117	1.03 × 10^9^
U3IHG8	Fructose-bisphosphate aldolase	39.27	8.74	−0.266	7.45 × 10^8^
R0KPC7	L-lactate dehydrogenase	36.75	7.73	−0.034	5.19 × 10^8^
R0L1Y3	60 kDa heat shock protein, mitochondrial	61.03	5.61	−0.069	4.98 × 10^8^
R0M210	Histone H2B	13.96	10.31	−0.738	4.65 × 10^8^
R0M714	Betaine--homocysteine S-methyltransferase	44.06	6.42	−0.348	4.35 × 10^8^

q-value is lower than 0.0001.

**Table 4:** Rank of the top 10 proteins of the highest signal strength in the PPSGP group.

Protein ID	Description	MW (kDa)	pI	GRAVY	iBAQ Value
P01989	Hemoglobin subunit alpha-A	15.44	8.54	0.068	4.12 × 10^9^
R0KA48	Histone H4	11.37	11.36	−0.521	1.83 × 10^9^
U3J1L1	Glyceraldehyde-3-phosphate dehydrogenase	35.84	8.87	−0.065	1.81 × 10^9^
U3J6E8	Apovitellenin-1	9.49	9.34	0.24	1.22 × 10^9^
R0KK84	ATP synthase subunit beta, mitochondrial	13.80	5.32	0.117	1.10 × 10^9^
U3IHG8	Fructose-bisphosphate aldolase	39.27	8.74	−0.266	1.03 × 10^9^
R0KPC7	L-lactate dehydrogenase	36.75	7.73	−0.034	5.63 × 10^8^
R0KFP7	Glutathione S-transferase	25.34	8.87	−0.319	5.37 × 10^8^
R0L1Y3	60 kDa heat shock protein, mitochondrial	61.03	5.61	−0.069	4.95 × 10^8^
A0A0K1R5T3	ADP/ATP translocase	32.79	9.73	0.092	4.64 × 10^8^

q-value is lower than 0.0001.

**Table 5:** Analysis of phosphorylated proteins.

Protein ID	Phosphorylation Site	Amino Acid	pI	GRAVY	iBAQ Value		
					GP	PSGP	PPSGP
U3J1L1	174	threonine	8.87	−0.065	1.50 × 10^9^	1.39 × 10^9^	1.81 × 10^9^
R0KFP7	37	threonine	8.87	−0.319	6.70 × 10^8^	3.82 × 10^8^	5.37 × 10^8^
A8RRQ6	237	serine	7.62	−0.326	1.83 × 10^8^	2.02 × 10^8^	2.91 × 10^8^
R0JSM9	263	serine	5.00	−0.710	9.51 × 10^7^	8.55 × 10^7^	9.34 × 10^7^
R0LAD1	4	threonine	9.78	−0.266	4.13 × 10^7^	3.31 × 10^7^	4.05 × 10^7^
R0LCM7	18	tyrosine	7.20	−0.399	1.08 × 10^7^	8.40 × 10^5^	5.74 × 10^6^
U3IQ39	20	serine	9.24	−0.975	8.70 × 10^6^	5.28 × 10^6^	4.27 × 10^6^
A0A068C605	7	threonine	9.39	0.027	7.02 × 10^6^	3.98 × 10^6^	5.51 × 10^6^
R0LFA3	61	threonine	5.61	−0.084	3.52 × 10^6^	3.83 × 10^6^	3.26 × 10^6^
U3IW62	110	lysine	8.91	−0.540	1.39 × 10^5^	/	/
U3IFD3	668	threonine	5.44	−0.877	/	5.22 × 10^4^	4.59 × 10^5^

q-value is lower than 0.0001.

## Data Availability

Data is contained within the article.

## References

[1] Wang L., Zou Y., Zhang K., Yu H. (2017). Ultrasonic-assisted alkaline extraction of duck liver protein and its antioxidant. Food Sci..

[2] Chen R., Zong Z.Q. (2013). Analysis of nutritional composition in muscle and liver of Lueyang black-bone chicken. Hubei Agr. Sci..

[3] Xia L.L., Wang Q.Q., Yang B., Sun X.X., Zhang Y.H., Liu L., Geng T.Y., Gong D.Q. (2016). Study on changes of blood chemistry indexes, hepatic routine nutritional composition and expression of lipid metabolism-associated genes during the recovery of geese with fatty livers. China Anim. Husb. Vet. Med..

[4] Huang F., Wu W. (2005). Antidiabetic effect of a new peptide from *Squalus mitsukurii* liver (S-8300) in streptozocin-induced diabetic mice. J. Pharm. Pharmacol..

[5] Liu Q., Li T. (2012). Study on anti-coagulation activity in Tibetan Medicine Yak liver protein in vitro. Lishizhen Med. Mater. Med. Res..

[6] Kristinsson H.G., Hultin H.O. (2003). Changes in conformation and subunit assembly of cod myosin at low and high pH and after subsequent refolding. J. Agric. Food Chem..

[7] Ingadottir B., Kristinsson H.G. (2010). Gelation of protein isolates extracted from tilapia light muscle by pH shift processing. Food Chem..

[8] Goto Y. (1989). Conformational states of β-lactamase: Molten-globule states at acidic and alkaline pH with high salt. Biochemistry.

[9] Wang Q., Jin Y., Xiong Y.L. (2018). Heating-aided pH shifting modifies hemp seed protein structure, cross-linking, and emulsifying properties. J. Agric. Food Chem..

[10] Goto Y., Calciano L.J., Fink A.L. (1990). Acid-induced folding of proteins. Proc. Natl. Acad. Sci. USA.

[11] Jiang S., Ding J., Andrade J., Rababah T.M., Almajwal A., Abulmeaty M.M., Feng H. (2017). Modifying the physicochemical properties of pea protein by pH-shifting and ultrasound combined treatments. Ultrason. Sonochem..

[12] Li J., Wu M., Wang Y., Li K., Du J., Bai Y. (2020). Effect of pH-shifting treatment on structural and heat induced gel properties of peanut protein isolate. Food Chem..

[13] Zhang W., Liu C., Zhao J., Ma T., He Z., Huang M., Wang Y. (2021). Modification of structure and functionalities of ginkgo seed proteins by pH-shifting treatment. Food Chem..

[14] Fu X.J., Lin Q.L., Li Z.H., Xu S.Y., Kim J.M. (2011). Effect of pH-shifting treatment on the Biochemical and thermal properties of myofibril protein. Adv. Mater. Res..

[15] Li X. (2018). Study on Extraction and Processing Properties of Goose Liver Proteins. Master’s Thesis.

[16] Sung H.Y., Chen H.J., Liu T.Y., Su J.C. (1983). Improvement of the functionalities of soy protein isolate through chemical phosphorylation. J. Food Sci..

[17] Moure A., Sineiro J., Dominguez H., Parajo J.C. (2006). Functionality of oilseed protein products: A review. Food Res. Int..

[18] Miedzianka J., Peksa A. (2013). Effect of pH on phosphorylation of potato protein isolate. Food Chem..

[19] Yin C.Y., Yang L., Zhao H., Li C.P. (2014). Improvement of antioxidant activity of egg white protein by phosphorylation and conjugation of epigallocatechin gallate. Food Res. Int..

[20] Xiong Z.Y., Ma M.H. (2017). Enhanced ovalbumin stability at oil-water interface by phosphorylation and identification of phosphorylation site using MALDI-TOF mass spectrometry. Colloids Surf. B Biointerfaces.

[21] Thaiphanit S., Anprung P. (2016). Physicochemical and emulsion properties of edible protein concentrate from coconut (*Cocos nucifera* L.) processing by-products and the influence of heat treatment. Food Hydrocoll..

[22] Kim N., Kwon D., Nam Y. (1988). Effects of phosphorylation and acetylation on functional properties and structure of soy protein. Korean J. Food Sci. Technol..

[23] Hu Z., Qiu L., Sun Y., Xiong H., Ogra Y. (2019). Improvement of the solubility and emulsifying properties of rice bran protein by phosphorylation with sodium trimetaphosphate. Food Hydrocoll..

[24] Chen G., Wang S., Feng B., Jiang B., Miao M. (2019). Interaction between soybean protein and tea polyphenols under high pressure. Food Chem..

[25] Jiang S., Zhang M., Liu H., Li Q., Xue D., Nian Y., Zhao D., Shan K., Dai C., Li C. (2022). Ultrasound treatment can increase digestibility of myofibrillar protein of pork with modified atmosphere packaging. Food Chem..

[26] Yan S., Xu J., Zhang S., Li Y. (2021). Effects of flexibility and surface hydrophobicity on emulsifying properties: Ultrasound-treated soybean protein isolate. LWT.

[27] Chen X., Zou Y., Han M., Pan L., Xing T., Xu X., Zhou G. (2016). Solubilisation of myosin in a solution of low ionic strength L-histidine: Significance of the imidazole ring. Food Chem..

[28] Maghamian N., Goli M., Najarian A. (2021). Ultrasound-assisted preparation of double nano-emulsions loaded with glycyrrhizic acid in the internal aqueous phase and skim milk as the external aqueous phase. LWT.

[29] Jorge S., Capelo J.L., LaFramboise W., Satturwar S., Korentzelos D., Bastacky S., Quiroga-Garza G., Dhir R., Wiśniewski J.R., Lodeiro C. (2021). Absolute quantitative proteomics using the total protein approach to identify novel clinical immunohistochemical markers in renal neoplasms. BMC Med..

[30] Hu H., Wu J., Li-Chan E.C.Y., Zhu L., Zhang F., Xu X., Fan G., Wang L., Huang X., Pan S. (2013). Effects of ultrasound on structural and physical properties of soy protein isolate (SPI) dispersions. Food Hydrocoll..

[31] Damodaran S., Nakai S., Modler H.W. (1996). Functional properties. Food Proteins-Properties and Characterization.

[32] Abdollahi M., Rezaei M., Jafarpour A., Undeland I. (2018). Sequential extraction of gel-forming proteins, collagen and collagen hydrolysate from gutted silver carp (*Hypophthalmichthys molitrix*), a biorefinery approach. Food Chem..

[33] Chen W., Wang W., Ma X., Lv R., Watharkar R.B., Ding T., Ye X., Liu D. (2019). Effect of pH-shifting treatment on structural and functional properties of whey protein isolate and its interaction with (−)-epigallocatechin-3-gallate. Food Chem..

[34] Jiang J., Xiong Y.L., Chen J. (2010). pH shifting alters solubility characteristics and thermal stability of soy protein isolate and its globulin fractions in different pH, salt concentration, and temperature conditions. J. Agric. Food Chem..

[35] Liu Y., Wang D., Wang J., Yang Y., Zhang L., Li J., Wang S. (2020). Functional properties and structural characteristics of phosphorylated pea protein isolate. Int. J. Food Sci. Technol..

[36] Sheng L., Ye S., Han K., Zhu G., Ma M., Cai Z. (2019). Consequences of phosphorylation on the structural and foaming properties of ovalbumin under wet-heating conditions. Food Hydrocoll..

[37] Jiang L.Z., Wang Z.J., Li Y., Meng X.H., Sui X.N., Qi B.K., Zhou L.Y. (2015). Relationship between surface hydrophobicity and structure of soy protein isolate subjected to different ionic strength. Int. J. Food Prop..

[38] Jambrak A.R., Mason T.J., Lelas V., Herceg Z., Herceg I.L. (2008). Effect of ultrasound treatment on solubility and foaming properties of whey protein suspensions. J. Food Eng..

[39] Broersen K., Van Teeffelen A.M.M., Vries A., Voragen A.G.J., Hamer R.J., De Jongh H.H.J. (2006). Do sulfhydryl groups affect aggregation and gelation properties of ovalbumin. J. Agric. Food Chem..

[40] Xiong Z., Zhang M., Ma M. (2016). Emulsifying properties of ovalbumin: Improvement and mechanism by phosphorylation in the presence of sodium tripolyphosphate. Food Hydrocoll..

[41] Yan C., Zhou Z. (2021). Solubility and emulsifying properties of phosphorylated walnut protein isolate extracted by sodium trimetaphosphate. LWT.

[42] Stanciuc N., Aprodu I., Rapeanu G., Bahrim G. (2012). Fluorescence spectroscopy and molecular modeling investigations on the thermally induced structural changes of bovine beta-lactoglobulin. Innov. Food Sci. Emerg..

[43] Ghobadi S., Ashrafi-Kooshk M.R., Mahdiuni H., Khodarahmi R. (2018). Enhancement of intrinsic fluorescence of human carbonic anhydrase II upon topiramate binding: Some evidence for drug-induced molecular contraction of the protein. Int. J. Biol. Macromol..

[44] Zhang Z.Y., Yang Y.L., Zhou P., Zhang X., Wang J.Y. (2017). Effects of high pressure modification on conformation and gelation properties of myofibrillar protein. Food Chem..

[45] Gong K.J., Shi A.M., Liu H.Z., Liu L., Hu H., Adhikari B., Wang Q. (2016). Emulsifying properties and structure changes of spray and freeze-dried peanut protein isolate. J. Food Eng..

[46] Xi C., Kang N., Zhao C., Liu Y., Sun Z., Zhang T. (2020). Effects of pH and different sugars on the structures and emulsification properties of whey protein isolate-sugar conjugates. Food Biosci..

[47] Liu J., Wan Y., Ren L., Li M., Lv Y., Guo S., Waqar K. (2021). Physical-chemical properties and in vitro digestibility of phosphorylated and glycosylated soy protein isolate. LWT.

[48] Zhao Q., Hong X., Fan L., Liu Y., Li J. (2022). Solubility and emulsifying properties of perilla protein isolate: Improvement by phosphorylation in the presence of sodium tripolyphosphate and sodium trimetaphosphate. Food Chem..

[49] Benelhadj S., Gharsallaoui A., Degraeve P., Attia H., Ghorbel D. (2016). Effect of pH on the functional properties of *Arthrospira* (*Spirulina*) *platensis* protein isolate. Food Chem..

[50] Lam R.S., Nickerson M.T. (2015). The effect of pH and temperature pre-treatments on the physicochemical and emulsifying properties of whey protein isolate. LWT.

[51] Diao X., Guan H., Zhao X., Chen Q., Kong B. (2016). Properties and oxidative stability of emulsions prepared with myofibrillar protein and lard diacylglycerols. Meat Sci..

[52] Xue S., Yu X., Li X., Zhao X., Han M., Xu X., Zhou G. (2019). Structural changes and emulsion properties of goose liver proteins obtained by isoelectric solubilisation/precipitation processes. LWT.

[53] Yu Y., Guan Y., Liu J., Hedi W., Yu Y., Zhang T. (2021). Molecular structural modification of egg white protein by pH-shifting for improving emulsifying capacity and stability. Food Hydrocoll..

[54] O’sullivan J., Murray B., Flynn C., Norton I. (2016). The effect of ultrasound treatment on the structural, physical and emulsifying properties of animal and vegetable proteins. Food Hydrocoll..

[55] Shi L., Beamer S.K., Yang H., Jaczynski J. (2018). Micro-emulsification/encapsulation of krill oil by complex coacervation with krill protein isolated using isoelectric solubilization/precipitation. Food Chem..

[56] Shao J.H., Zou Y.F., Xu X.L., Wu J.Q., Zhou G.H. (2011). Evaluation of structural changes in raw and heated meat batters prepared with different lipids using Raman spectroscopy. Food Res. Int..

[57] Li-Chan E., Nakai S., Wood D.F. (1984). Hydrophobicity and solubility of meat proteins and their relationship to emulsifying properties. J. Food Sci..

